# Adverse effects of long-term Levothyroxine therapy in Subclinical Hypothyroidism

**DOI:** 10.1016/j.amsu.2022.103503

**Published:** 2022-04-04

**Authors:** Sidhant Ochani, Amna Siddiqui, Alishba Adnan

**Affiliations:** a Khairpur Medical College, Pakistan; b Karachi Medical and Dental College, Pakistan

## Abstract

Levothyroxine, also known as L-thyroxine, is a manufactured form of the thyroid hormone thyroxine which is considered to be a gold-standard therapy for treating an underactive thyroid gland (hypothyroidism). Some experts highlight benefits of LT4 replacement therapy, others specify the harmful effects of it, in regards to treating subclinical hypothyroidism. With respect to current guidelines for the consistent dosing of levothyroxine for treating hypothyroidism, we aim to highlight limitations of long-term use of Levothyroxine therapy in SCH. Apropos of the studies published to date, we look into the appropriate dosing of Levothyroxine in SCH by physicians.

Respected Editor

Levothyroxine, also known as l-thyroxine, is a manufactured form of the thyroid hormone thyroxine which is considered to be a gold-standard therapy for treating an underactive thyroid gland [1] (hypothyroidism). With respect to current guidelines for the consistent dosing of levothyroxine for treating hypothyroidism, we aim to highlight limitations of long-term use of Levothyroxine therapy in SCH. Apropos of the studies published to date, we look into the appropriate dosing of Levothyroxine in SCH by physicians.

Subclinical hypothyroidism (SCH) is defined as a serum thyroid-stimulating hormone (TSH) level (4.6–10 mlU/L) above the upper limit, despite normal levels of serum free thyroxine. In Pakistan, SCH prevails up to 4.1% in adults, and 5.4% in children and is more inclined towards females [2].

Recent studies by Brito et al., show that use of Levothyroxine in nonpregnant adults with SCH (thyrotropin level elevated but ≤10 mIU/L and normal free thyroxine [FT4] levels), demonstrate no clinically relevant benefits of levothyroxine replacement for quality of life or thyroid-related symptoms [3]. It is known to have an increased risk of cancer in both males and females [4] but is still used for its beneficial effects to the patients of hypothyroidism.

The studies proving this are not limited in numbers, they demonstrate an association between long-term levothyroxine therapy and increased risk of heart disease, osteoporosis, and fractures. In addition to this it also increases the economic burden on the patients owing to price surge [5]. Conclusively, long-term dosing of Levothyroxine does not provide any benefit, but it can predict the harm [[6], [7], [8]].

This is controversial essentially, some experts highlight benefits of LT4 replacement therapy, others specify the harmful effects of it, in regards to treating subclinical hypothyroidism. Therefore, the time has come to stop overuse of levothyroxine in patients with SCH by keeping in consideration proper guidelines & recommendations [9] which should be evaluated together with the clinical judgement of physicians up to present time in order to give the best care.

## Ethical approval

N/A.

## Sources of funding

None.

## Author contributions

Sidhant Ochani: Conceptualized the topic, did the literature review, wrote the first draft, did the referencing.

Amna Siddiqui: Wrote the first draft, did the literature review.

Alishba Adnan: Reviewed, edited and wrote the final draft.

## Registration of research studies

N/A.

## Guarantor

Sidhant Ochani.

## Consent

N/A.

## Address

All Authors belong to Karachi, Sindh, Pakistan.

## Declaration of competing interest

None.
